# Shear bond strength evaluation of metallic brackets bonded to a CAD/CAM PMMA material compared to traditional prosthetic temporary materials: an in vitro study

**DOI:** 10.1590/2177-6709.25.3.031-038.oar

**Published:** 2020

**Authors:** Gonzalo Andrés Garcés, Victor Hugo Rojas, Cristian Bravo, Camila S. Sampaio

**Affiliations:** 1 Universidad de Los Andes, Faculty of Dentistry, Department of Biomaterials (Santiago, Chile).; 2 Universidad de Los Andes, Faculty of Dentistry, Department of Orthodontics (Santiago, Chile).

**Keywords:** Air abrasion, Bonding agents, CAD/CAM, Orthodontics, Acrylic resins

## Abstract

**Introduction:**

Orthodontic treatment for adults is currently increasing, and therefore the need to bond brackets to restorations and temporary crowns. The use of CAD/CAM PMMA provisional restorations for orthodontic purposes have not yet been described, and there is currently insufficient information regarding the strength of bracket adhesion.

**Objective:**

This study aimed at evaluating the effects of thermocycling (TC) and surface treatment on shear bond strength (SBS) of brackets to different provisional materials.

**Methods:**

Forty specimens were made from each material [PMMA (Telio Lab), bis-acryl (Telio CS C&B), and PMMA CAD/CAM (Telio CAD)], sandpapered, and divided according to surface treatment (pumiced or sandblasted) and TC (half of the samples = 1,000 cycles, 5°C/55°C water baths) (n = 10/group). Stainless-steel brackets were bonded to the specimens (using Transbond XT), and SBS testing was performed. Data were analyzed by three-way ANOVA and LSD *post-hoc* tests (α = 0.05). Failure types were classified with adhesive remnant index (ARI) scores.

**Results:**

SBS values ranged from 1.5 to 14.9 MPa. Sandblasted bis-acryl and sandblasted auto-curing PMMA groups presented similar values (*p*> 0.05), higher than the CAD/CAM material (*p*< 0.05), with or without TC. When thermocycled, pumiced bis-acryl showed higher SBS than pumiced acrylic (*p*= 0.005) and CAD/CAM materials (*p*= 0.000), with statistical difference (*p*= 0.009). TC showed negative effect (*p*< 0.05) for sandblasted bis-acryl and pumiced acrylic groups. ARI predominant score was mostly zero (0) for CAD/CAM, 1 and 2 for bis-acryl, and 1 for acrylic groups.

**Conclusion:**

In general, bis-acryl material showed the highest SBS values, followed by acrylic and CAD/CAM materials, which showed SBS values lower than an optimum strength for bonding brackets.

## RESUMO

**Introdução:**

Atualmente, a demanda por tratamento ortodôntico em adultos tem aumentado. Consequentemente, também tem aumentado a necessidade de se colar braquetes em restaurações e coroas provisórias. O uso de restaurações provisórias de PMMA CAD/CAM com finalidade ortodôntica ainda não foi descrito e, até a presente data, não há informação suficiente sobre a resistência da colagem dos braquetes a esse tipo de material.

**Objetivo:**

O presente estudo teve como objetivo avaliar os efeitos da termociclagem (TC) e do tratamento de superfície sobre a resistência da colagem ao cisalhamento (RC) de braquetes colados em diferentes materiais provisórios.

**Métodos:**

Quarenta espécimes foram confeccionados de cada material: resina acrílica PMMA (Telio Lab), resina bisacrílica (Telio CS C&B), e PMMA CAD/CAM (Telio CAD). Em seguida, foram lixados e divididos de acordo com o tratamento de superfície (polidos ou jateados) e TC (metade da amostra - 1.000 ciclos de imersão em água a 5°C e 55°C) (n = 10/grupo). Braquetes de aço inoxidável foram colados aos espécimes (utilizando Transbond XT) e realizou-se o teste de RC. As informações foram analisadas por meio dos testes ANOVA de três vias e *post-hoc* LSD (α?#8197;= 0,05). Os tipos de falha foram classificados de acordo com os escores do índice de adesivo remanescente (ARI).

**Resultados:**

Os valores de RC variaram de 1,5 a 14,9 MPa. Os grupos de resina bisacrílica jateada e de PMMA autopolimerizável jateado apresentaram valores semelhantes (*p*> 0,05), superiores ao do material CAD/CAM (*p*< 0,05) com ou sem TC. Quando submetido à TC, o grupo de resina bisacrílica polida apresentou resistência ao cisalhamento maior do que os grupos de resina acrílica polida (*p*= 0,005) e do material CAD/CAM (*p*= 0,000), com diferença estatística (*p*= 0,009). A TC apresentou efeito negativo (*p*< 0,05) para os grupos de resina bisacrílica jateada e de resina acrílica polida. O escore ARI = 0 foi predominante para o grupo CAD/CAM; os escores 1 e 2, para o grupo resina bisacrílica; e o escore 1, para o grupo resina acrílica.

**Conclusão:**

De forma geral, a resina bisacrílica apresentou os maiores valores de RC, seguida da resina acrílica e do material CAD/CAM, que apresentaram valores de RC inferiores à força recomendada para colagem de braquetes.

## INTRODUCTION

The search for orthodontic treatments by adults is currently increasing, not only because of esthetics, but also because of frequently being an intermediate stage on oral rehabilitation.^1^ Facing this new scenario, orthodontists often need to bond brackets to restorations and temporary crowns, since adult dentition is usually characterized by restorative treatments.^2^ An issue regarding this procedure relies on the fact that bonding brackets to restorative materials is claimed to be more difficult than to natural teeth.^2^ ^,^ ^3^ Although not many studies have been performed on provisional restorations,^2^ ^-^ ^8^ some showed less than the minimum bonding necessary to be able to perform tooth movement in the orthodontic treatment.^2^ ^,^ ^3^

Factors including physical, mechanical, handling properties and biocompatibility might influence the choice of a material for provisional restorations,^1^ which should work as protection of the pulpal tissues, and present esthetics and oral functions.^9^

Traditionally, provisional restorations are made from auto-curing polymethylmethacrylate (PMMA) resins.^6^ However, these are prone to discoloration and can cause chemical irritation or allergic reactions during polymerization.^10^ Also, their polymerization shrinkage can cause marginal discrepancies in the provisional crowns.^11^ A new class of material, the bis-acryl resins, shows low exothermic reaction during setting, with better strength, marginal adaptation and contour.^12^ Another class of provisional material involves a novel technique, the computer-aided design/computer-aided manufacturing (CAD/CAM) materials. The emergence of this technology allowed for high precision materials, since restoration is milled from pre-polymerized blocks of the provisional material, thus, any degree of polymerization shrinkage occurs during processing of the block and not intra-orally.^9^ Moreover, this indirect material presents higher fracture strength and lower marginal gap than direct techniques such as bis-acryl resins.^9^ However, one disadvantage is the cost, in comparison to conventional provisional restorations.^9^ The use of CAD/CAM PMMA provisionals for orthodontic purposes had not yet been described in literature.

Although a well-known technique has been used to bond brackets to natural teeth, when it comes to provisional materials, no technique has been specifically described. A strong bond of composite to enamel has been possible since the introduction of the use of phosphoric acid in dentistry, by Buonocore.^13^ Since then, different techniques have been studied to improve bond strength of brackets to different surfaces, such as sandblasting or air abrasion,^14^ pulsed lasers^15^ and surface roughening with a bur.^5^

A clinically acceptable adhesive resistance for bracket bonding has been claimed to vary from 6 to 8 MPa,^16^ ^,^ ^17^ where brackets bonded to provisional materials must be strong enough to resist dental movement, but weak enough to be removed without damaging the bonded surface when treatment is finished. Excessive bond strength is undesirable, since it does not allow for smooth debonding, without damaging the restorative surface.^18^

In order to evaluate bonding of brackets to different surfaces *in vitro*, thermocycling can be used as an accelerated aging test.^19^ Temperature changes between the water baths could contribute to water contamination at the resin bond interface, thus weakening the resin.^20^

Therefore, the aim of this study was to evaluate the effect of thermocycling (TC) and surface treatment on shear bond strength of metallic brackets to different provisional prosthetic materials. The hypothesis tested was that different provisional materials present different bond strengths to metallic brackets. The use of different surface treatments might result in different shear bond strengths; and TC results in lower bond strengths compared to groups without TC.

## MATERIAL AND METHODS

**Figure 1:**
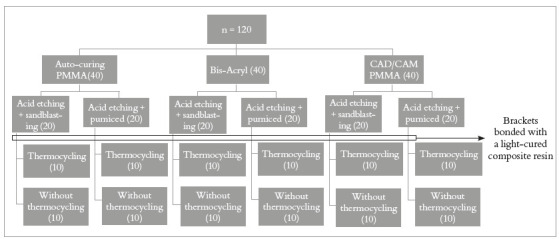
Illustration of the groups’ setting process.

One hundred and twenty samples were fabricated according to the provisional prosthetic material used, surface treatment and TC (n = 10 per group). Group setting can be seen on Figure 1. Forty cylindrical specimens (7-mm diameter x 2-mm thick) were made from each material, according to manufacturer’s instructions: PMMA auto-curing acrylic resin (Telio Lab, Ivoclar Vivadent, Schaan, Liechtenstein) and bis-acrylic resin (Telio CS C & B, Ivoclar Vivadent, Schaan, Liechtenstein). Regarding the CAD/CAM PMMA material (Telio CAD, Ivoclar Vivadent, Schaan, Liechtenstein), blocks were cut (6mm x 8mm x 2mm thick) with a slow speed diamond saw (Mecatone T180, Presi, Eybens, France). Surfaces from all materials were polished with 120-, 500- and 1000-grit SIC paper discs respectively, for 20s (each grit) (Labopol-6, Struers, Westlake, Ohio, USA), being washed and cleaned in between discs. 

**Figure 2:**
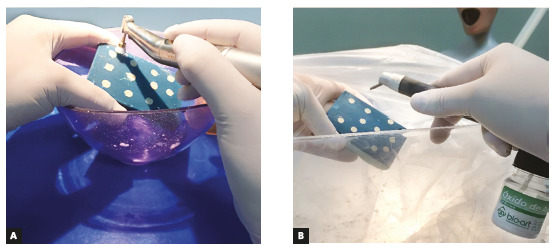
Photographs of the surface treatment process: A) surfaces polishing with pumice slurry; B) sandblasting of the samples with 50-µm Al_2_O_3_ particles.

**Figure 3:**
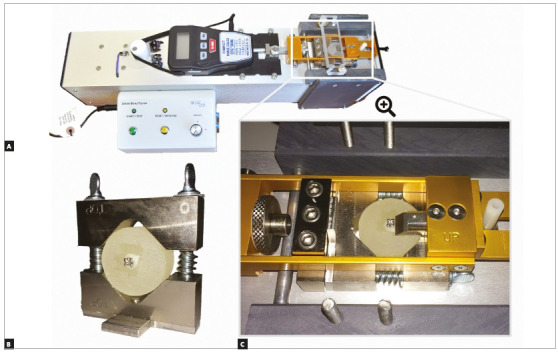
A) Image showing the Shear Bond Tester^®^ device in a panoramic view. In (C), it is possible to observe a photographic close-up of the area where the machine generates the shear force on the bracket, which is bonded to a cylindrical sample of provisional prosthetic material, until the adhesive failure occurs. In (B), it is possible to observe the test object, which is held by the specially designed support of the machine.

After that, samples from each material were randomly and equally subdivided into two groups regarding surface treatment: pumice slurry on a prophylaxis brush for 5 seconds or sandblasting (50- µm Al_2_O_3_ particles at 10-mm distance for 5 seconds - Microjato Standard, BioArt, São Carlos/SP, Brazil). Following this procedure, all prosthetic surfaces underwent acid etching with phosphoric acid for 30s, in order to clean the samples’ surfaces and remove any possible oil or debris from the sample-making process. Maxillary central incisors brackets with a micro-etched 80-gauge mesh base (Gemini, 3M Unitek, Monrovia, California, USA) were bonded with a light-cured composite resin (Transbond XT, 3M Unitek, St. Paul, MN, USA) according to manufacturer’s instructions, using a LED light-curing unit (Bluephase Style 20i, IvoclarVivadent, Schaan, Liechtenstein). This procedure was performed by a single operator in order to standardize the steps (Fig 2). Then, samples were stored in a controlled atmosphere at 100% humidity for 24h. After that, half of the samples from each group were subjected to a shear bond strength (SBS) test at a cross-head speed of 0.5mm/min until failure, in a shear bond strength tester (Shear Bond Tester, Bisco Dental, Portland, OR, USA) (Fig 3). The other half of the samples underwent a TC procedure applying 1,000 cycles of alternating 5^o^C and 55^o^C water baths (30s each), followed by the SBS test procedure, as previously described. Although ISO/TR 11405^21^ recommends 500 cycles as a methodology for aging studies, a lack of difference between groups when using this amount of cycles has been observed, which is the reason why, in the present study, this value was doubled. SBS values were obtained in Newtons and converted to MPa.

After debonding, each specimen was analyzed under a loupe (Panoramic Flip-up Adivista 2.5x; PeriOptix Inc., Lompoc, CA, USA) at 2.5 times magnification, to evaluate failure, described through the adhesive remnant index (ARI)^22^: ARI 0 (0% on sample, 100% on bracket), ARI 1 (<50% on sample, >50% on bracket), ARI 2 (>50% on sample, <50% on bracket), or ARI 3 (100% on sample, 0% on bracket).

SBS data were analyzed using three-way analysis of variance and compared with LSD’s *post-hoc* tests, at α = 0.05. 

## RESULTS

Table 1 summarizes the mean and standard deviation of SBS determined for each material, according to surface treatment and TC. A significant statistical difference was found among groups (*p*< 0.05). SBS values ranged from a minimum of 1.5 MPa (pumiced CAD/CAM material with TC), to a maximum of 14.9 MPa for the sandblasted bis-acryl material without thermocycling.

**Table 1:** Means (SD) of different materials regarding surface treatment and thermocycling (values in MPa).

TC	Material	Treatment	
		Sandblasted	Pumiced
Without	CAD-CAM	3.2 (1.6)^Ba^	4.0 (2.0)^Ba^
	Bisacryl	14.9 (4.2)^Aa^	13.9 (5.3)^Aa^
	Acrylic	13.7 (3.6)^Aa^	11.1 (2.1) ^Aa^
With	CAD-CAM	2.7 (2.3)^Ba^	1.5 (0.8)^Ca^
	Bisacryl	11.4 (4.1)^Aa^*	11.4 (5.1)^Aa^
	Acrylic	12.1 (4.7)^Aa^	6.2 (3.5)^Bb^*

Means followed by different letters (uppercase in vertical and lowercase in horizontal) differ from each other (p < 0.05) within the same group of cycling.

* Differs from before and after thermocycling in the same material and surface treatment (p < 0.05).

The bis-acryl material showed the highest SBS values when compared to other materials, regardless of the type of treatment or TC, while the CAD/CAM material showed the lowest values. When no TC was performed, the bis-acryl and the acrylic materials were not significantly different from each other for any treatment (*p*> 0.05), although both materials showed higher SBS values when compared to the CAD/CAM material, for any treatment (*p*< 0.05). When no TC was performed, both sandblasting or pumiced treatments performed statistically similar for all materials (*p*> 0.05); while when TC was performed, the acrylic material showed a statistically significant difference (*p*= 0.001), favoring the sandblasted, compared to the pumiced group, which was almost twice the value, when compared.

When TC was performed, sandblasted bis-acryl and acrylic materials performed statistically similar (*p*= 0.656), both showing higher SBS values than the CAD/CAM material (*p*< 0.05). However, when TC and pumice were performed, the bis-acryl material was statistically superior to the acrylic (*p*= 0.003) and CAD/CAM (*p*= 0.000) materials, followed by the acrylic material and lastly the CAD/CAM material, also statistically different within each other (*p*= 0.009). 

When TC was compared to without-TC, the only groups that showed a statistically significant difference were the sandblasted bis-acryl material (*p*= 0.030), that showed higher SBS values when no TC was performed, and the pumiced acrylic material, showing the same pattern (*p*= 0.005).

**Figure 4:**
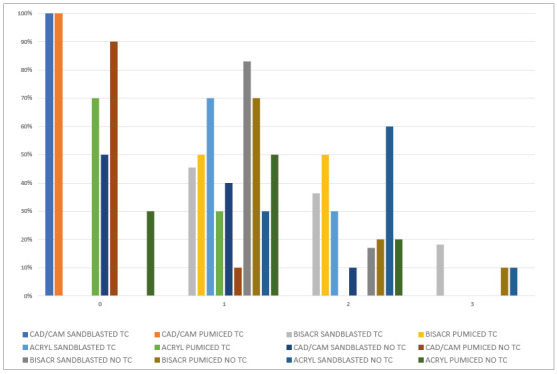
Results from the Adhesive Remnant Index (ARI) per group.

Figure 4 shows the results from the Adhesive Remnant Index (ARI) types for all groups. It was noted that failures were different for each material, being a predominant ARI = 0 for the CAD/CAM groups, a predominant ARI = 1 and ARI = 2 for the bis-acryl groups, and ARI = 1 for the acrylic groups. It was observed that, for the bis-acryl, a great amount of specimens showed failure within the sample, meaning that the adhesion was so strong that instead of fracturing the adhesive interface, the specimen cohesively fractured, thus detaching the bracket. This did not occur with the CAD/CAM material, and rarely happened with the acrylic material.

## DISCUSSION

This study evaluated SBS of brackets using a novel material technology for provisional restorations - results that have not been studied so far. Results from this study showed that significant changes occur on bond strength when independent variables, such as materials, surface treatment and thermocycling, are tested.

The first tested hypothesis was accepted, since different materials promoted different SBS to metallic brackets. In general, the bis-acryl material promoted the higher SBS values when the different materials were compared, although for most of the groups it performed similar to the acrylic resin. The acrylic material showed lower SBS values than the bis-acryl material when both groups were pumiced and TC was performed, which is in agreement with a previous study.^6^ However, sandblasting produced statistically similar results for both materials, which is also in agreement with a previous study.^23^ When the CAD/CAM PMMA provisional material was evaluated, it showed the lowest SBS values, independent of surface treatment and TC - a material that has not been studied so far for brackets bonding.

The higher SBS of bis-acryl and acrylic materials, compared to the CAD/CAM material, can be explained because their basic components are methacrylates; thus, bonding is likely to be influenced by the number of available reactive sites on the polymerized provisional materials.^3^ Moreover, the bis-acryl material contains bifunctional acrylates, with available bonding sites and cross-link to provide increased mechanical strength and resistance to weakening in the presence of water.^24^ On the other hand, PMMA CAD/CAM blocks are pre-polymerized blocks, and thus, a material with greater density and fewer potential bonding sites, the same process that occurs in traditional denture teeth,^23,25^ contributing to their low SBS to metallic brackets. Although this material presents a high fracture strength, low polymerization shrinkage, and excellent marginal adaptation,^9^ this study showed that it is not indicated for orthodontic movements applied to a provisional restoration, due to the unacceptable SBS results. 

Although ARI score remained almost absolutely equal to 0 in the CAD/CAM materials for all groups, no surface damage was observed, meaning that adhesion was poor and promoted total debonding of bracket/resin from the sample, while the material resisted to shear bond forces. On the other hand, most of the bis-acryl material’s samples showed a degree of restoration surface damage, correlated to the high bond strength of this material to the orthodontic cement. Although damage occurred, an important characteristic regarding this material relates to its ease of repair with a composite resin.^23,26,27^ Moreover, it possesses other advantages over acrylic resins, such as superior handling characteristics, ease of manipulation, less porosity, low polymerization shrinkage and good color stability.^23^ ^,^ ^26^ ^,^ ^27^

When surface treatment was compared, a significant difference was observed only for the acrylic group after TC, with pumiced group presenting lower SBS values than sandblasted group. Thus, the second hypothesis was partially accepted. The difference was twice the value, meaning that sandblasting is indicated for the long-term success of bracket bonding to acrylic provisionals. Moreover, results from pumiced acrylic restorations after TC performed at the exact acceptable value for bracket bonding; thus, it is likely that over time those values could decrease to an unacceptable point. There is an increase of SBS for brackets bonded to sandblasted polycarbonate crowns, while non-sandblasted (control) crowns produced statistically lower SBS,^5^ which is in accordance with the present study. When the ARI was evaluated, for all materials, more frequent ARI = 0 values were observed when pumiced was compared to sandblasted. An ARI = 0 represents that no orthodontic composite remained adhered to provisional material, possibly due to the high adhesion values between the bracket and the adhesive system,^28^ and thus lower adhesion values between the orthodontic composite and the provisional material. An exception is made for the bis-acryl material, where both sandblasted and pumiced groups behaved similarly and ranging mostly from ARI = 1 to ARI =2, for both with and without TC. The present study used a magnification of 2.5x to evaluate the ARI of all samples; however, it has been described that ARI scores observed through different magnifications (from 10x to 20x) can present significantly different results.^29^ Thus, future studies should focus on the visualization of ARI scores with increased magnification.

The third hypothesis was also partially accepted, since two groups presented a statistical difference when compared before and after TC. Sandblasted bis-acryl material presented significantly higher SBS values when no TC was performed, compared to when it was. However, both values were considered way above the acceptable threshold for brackets bonding, which are usually considered from 6 to 8 MPa.^16,17^ The same pattern occurred for the pumiced acrylic material; however, when this group underwent TC, SBS values remained at the exact value of tolerance for a bracket bonding to be acceptable. Thus, likely with longer aging procedures, it would present values below the acceptable limits. Therefore, when acrylic materials are used for orthodontic purposes, sandblasting is highly indicated. 

Results from this study suggest that bis-acryl and acrylic materials should be preferred against the CAD/CAM material, when bonding of brackets are to be performed within the tested conditions. However, for the acrylic materials, sandblasting is paramount for achieving positive long-term results. Possible solutions regarding improving brackets adhesion to CAD/CAM PMMA materials should be studied, such as promoting micromechanical retentions in order to increase surface area, or by using silane coupling agents, which has shown to improve shear bond strength between resin composite cements and different materials such as ceramics.^30^

This study followed the manufacturer’s recommendation on brackets cementation with the system used; however, further studies should focus on longer aging times and, mostly, on using extra adhesive steps or different surface treatments, in order to improve bracket adhesion to CAD/CAM PMMA materials, as this material is being increasingly used in dentistry, showing good properties regarding marginal fracture strength, low marginal gap, and no polymerization shrinkage in mouth.^9^

## CONCLUSIONS

Within the limitations of this *in vitro* study, it can be concluded that the evaluated bis-acryl material showed the highest shear bond strength results when all variables were considered, although when sandblasting was performed, values for this material and the acrylic resin remained statistically similar, both with or without TC. If the auto-curing acrylic resin is the material of choice for the provisional restoration, the orthodontist should sandblast the provisional restoration before bracket adhesion, in order to obtain longer successful results. The PMMA CAD/CAM material showed an insufficient SBS to metallic brackets within the tested conditions.
